# Posttraumatic stress disorder and diabetes-related outcomes in patients with type 1 diabetes

**DOI:** 10.1038/s41598-023-28373-x

**Published:** 2023-01-27

**Authors:** Frederike Lunkenheimer, Alexander J. Eckert, Dörte Hilgard, Daniel Köth, Bernhard Kulzer, Ursula Lück, Blanca Lüdecke, Antonia Müller, Harald Baumeister, Reinhard W. Holl

**Affiliations:** 1 Department of Clinical Psychology and Psychotherapy, Institute of Psychology and Education, Ulm University, Lise-Meitner-Str. 16, 89081 Ulm, Germany; 2 Institute of Epidemiology and Medical Biometry, ZIBMT, Ulm University, Albert-Einstein-Allee 41, Ulm, Germany; 3 German Center for Diabetes Research (DZD), Ingolstädter Landstraße 1, Munich-Neuherberg, Germany; 4 Pediatric Endocrinology and Diabetology, Primary Psychosomatic Care, Bahnhofstraße 54, Witten, Germany; 5 Department of Endocrinology and Diabetology, Hospital Sachsenhausen, Schulstraße 31, Frankfurt, Germany; 6 Research Institute of the Diabetes-Academy Mergentheim (FIDAM), Theodor-Klotzbücher-Straße 12, Bad Mergentheim, Germany; 7 Department of Pediatrics and Adolescent Medicine, Regional Hospital Mödling, Sr. M. Restituta-Gasse 12, Mödling, Austria; 8 Diabetes Centre, Alexianer St. Hedwig Hospital, Große Hambuger Straße 5-11, Berlin, Germany; 9 Clinic Group Dr. Guth GmbH & Co. KG, Clinical Center Karlsburg, Greifswalder Straße 11, Karlsburg, Germany

**Keywords:** Metabolic disorders, Psychiatric disorders, Trauma, Psychology, Health care, Medical research

## Abstract

Mental comorbidities in patients with type 1 diabetes mellitus (T1D) are common, and can have a negative impact on acute blood glucose levels and long-term metabolic control. Information on the association of T1D and comorbid posttraumatic stress disorder (PTSD) with diabetes-related outcomes is limited. The aim was to examine the associations between a clinical diagnosis of PTSD and diabetes-related outcomes in patients with T1D. Patients with T1D and comorbid documented PTSD from the DPV database (n = 179) were compared to a group with T1D without PTSD (n = 895), and compared to a group with T1D without comorbid mental disorder (n = 895) by matching demographics (age, gender, duration of diabetes, therapy and migration background) 1:5. Clinical diabetes-related outcomes {body mass index (BMI), hemoglobin A1c (hbA1c), daily insulin dose, diabetic ketoacidosis (DKA), hypoglycemia, number of hospital admissions, number of hospital days} were analyzed, stratified by age groups (≤ 25 years vs. > 25 years). Patients with comorbid PTSD aged ≤ 25 years compared with patients without PTSD or patients without mental disorders had significantly higher HbA1c (8.71 vs. 8.30 or 8.24%), higher number of hospital admissions (0.94 vs. 0.44 or 0.32 per year) and higher rates of DKA (0.10 vs. 0.02 or 0.01 events/year). Patients with comorbid PTSD aged ≤ 25 years compared with patients without PTSD had significantly higher BMI (0.85 vs. 0.59) and longer hospital stays (15.89 vs.11.58 days) than patients without PTSD. Patients with PTSD > 25 years compared with patients without PTSD or without any mental comorbidities had significantly fewer hospital admissions (0.49 vs. 0.77 or 0.69), but a longer hospital length of stay (20.35 vs. 11.58 or 1.09 days). We found that PTSD in younger patients with T1D is significantly related to diabetes outcome. In adult patients with T1D, comorbid PTSD is associated with fewer, but longer hospitalizations. Awareness of PTSD in the care of patients with T1D should be raised and psychological intervention should be provided when necessary.

## Introduction

Over 14% of people with type 1 diabetes (T1D) have a comorbid mental disorder, such as anxiety, eating, mood, personality and behavior disorder. This is twice the number compared to controls without T1D^1^. Most studies focused on common comorbid mental disorders particularly depression and anxiety^1,2^ with only limited information on the association between T1D and posttraumatic stress disorder (PTSD).

According to the DSM-5^3^, PTSD is a trauma- and stressor-related disorder caused by a traumatic event and is defined by four characteristic symptom domains including persistent re-experience, avoidance, negative mood and cognitions, and hyperarousal, causing clinically significant distress and/or functional impairment. Results from the World Mental Health Surveys^4^ indicate a point prevalence of PTSD in the general population of 1.4%. Studies on pediatric populations reported prevalence rates of PTSD or clinically relevant posttraumatic stress symptoms (PTSS) in patients with T1D from 5 to 19%^5,6^. Among adults with T1D, the prevalence rate for PTSD regardless of trauma type is 0.5%^2^ and for PTSS when considering hypoglycemic episode as a potentially traumatic event is 25%^7^. The prevalence rates in patients with T1D and comorbid PTSD or PTSS compared to the general population, indicate an increased rate of comorbidity in T1D. However, there is little research on people with T1D and PTSD, as studies predominantly focused on type 2 diabetes (T2D) or other chronic somatic diseases and comorbid PTSS or PTSD (e.g. Arigo et al.^8^).

T1D and PTSD can have reciprocal effects. T1D is a chronic condition and, considering the aversive experience of diagnosis and medical treatment, could be experienced as a traumatic event and thus be the trigger for PTSD^3^. On the other hand, people with PTSD may have metabolic and neuroendocrine dysfunction as well as alterations in inflammatory pathways, which may not be found in people without PTSD^9–11^. In addition to a pathophysiology, PTSD is associated with poorer health behavior, which may be risk factors for developing CD and may negatively affect CD outcome^12–16^. For example, in addition to challenges of T1D in children, adolescents and young adults (CAYA), mental comorbidities such as depressive symptoms and anxiety disorders lead to additional difficulties that can overwhelm daily medical care and manifest in poor diabetes-related outcomes, such as worse glycemic control (higher HbA1c levels) and more frequent diabetic ketoacidosis (DKA), compared to CAYA with T1D without mental comorbidities^17–19^. In adulthood, study results are heterogeneous. Van Bastelaar et al. study results show that comorbidity of mental disorders in adults with diabetes (T1D and T2D) is also associated with poor treatment adherence, poor glycemic control (elevated HbA1c levels), increased emergency admissions for DKA and more frequent hospitalizations^20^. On the other hand, Subasinghe et al. could not find an association between a comorbid mental disorder such as a mood disorder in adults with T1D and worse diabetes-related outcomes^21^. The psychological distress induced by a mental comorbidity could negatively affect acute glucose levels and long-term metabolic control, and impair the patient's ability to engage in healthy self-care behaviors^22,23^. The special characteristic of PTSD compared to other mental diseases is its ascertainable etiology^3,24^. Especially if PTSD is triggered by a medical event related to T1D, it can negatively impact medication and treatment adherence. Treatment for T1D may serve as an aversive reminder, which in turn may increase avoidance behavior characteristic of PTSD^25–27^. Information on the association of patients with T1D and comorbid PTSD with diabetes-related outcomes is limited.

## Objective

The aim of this study was to examine the association between PTSD and relevant diabetes-related outcomes in people with T1D and comorbid PTSD compared to a matched control group with T1D without PTSD and compared to another matched control group with T1D without mental disorder. In addition, we investigated whether there is a difference in diabetes-related outcomes by age group.

## Method

### Data selection and data extraction

The German diabetes follow-up registry (Diabetes-Patienten-Verlaufsdokumentation, DPV), which is a standardized electronic health record developed at the Institute of Epidemiology and Medical Biometry, ZIBMT, Ulm University, Germany, collects data from patients with all types of diabetes. The approval of the Ethics Committee of the University of Ulm for data collection and analysis of anonymized data was obtained (approval number: 202/09). Patient data come from a total of 511 centers. Of these, 463 are from Germany, 46 from Austria, four from Switzerland and one from Luxembourg. Data collection started in 1995 and data until April 2022 were included into this analysis.

Migration background was defined as the patient or at least one parent born outside a country of the DPV centers (Germany, Austria, Switzerland, or Luxembourg). Body mass index (BMI) was reported in the group ≤ 25 years based on reference values from Arbeitsgemeinschaft Adipositas im Kindes- und Jugendalter (AGA) using Cole's LMS method^28,29^. Hemoglobin A1c (HbA1c) values were transformed by the “multiple of the mean” method (MOM) to the Diabetes Control and Complications Trial (DCCT) reference range of 4.05–6.05% (20.7–42.6 mmol/mol)^30^. Diabetic ketoacidosis (DKA) was defined as pH < 7.3 and/or bicarbonate < 15 mmol/mol according to the guidelines of International Society for Pediatric and Adolescent Diabetes (ISPAD)^31^. Severe hypoglycemic events were defined as hypoglycemic situations in which the patients required assistance from other persons and severe hypoglycemia with coma was defined by unconsciousness or convulsion. Patients included in the DPV initiative were enrolled in this study if they had a clinical diagnosis of T1D. All patients with T1D at all ages were further divided into groups for analysis. Patients were assigned to the *T1D* + *PTSD* group if the terms “posttraumatic stress disorder”, “PTSD” or codes of International Classification of Diseases—Tenth Edition (ICD-10) “F43.1” or Diagnostic and Statistical Manual of Mental Disorders—Fifth Edition (DSM-V) “309.81” were documented at least once during the clinical course of the patients. Patients with T1D and other comorbid mental health diagnoses documented in addition to PTSD were not excluded from the T1D + PTSD group. The criterion for the T1D + PTSD group was thus a PTSD diagnosis, regardless of whether other mental health comorbidities were present or not. Patients with T1D who had no documentation of PTSD formed the *T1D-no-PTSD* group, regardless of whether another mental disorder was present or not. Patients with T1D who had no documentation of PTSD and no evidence of any other mental disorder (depression, anxiety/compulsive disorder, attention and activity disorder, eating disorder, borderline personality disorder, schizophrenia, autism, suicidality and non-suicidal self-injury behavior) formed the *T1D-only* group. Data of demographic and diabetes-related outcomes were aggregated for the most recent treatment year per patient.

### Data analysis

All statistical analyses were generated using SAS (Statistical Analysis Software, SAS Institute Inc., Cary, NC, USA) Version 9.4 build TS1M7 on a Windows server 2019 mainframe. Demographic data and diabetes-related outcomes were analyzed descriptively. Results were presented as median with lower (Q1) and upper quartiles (Q3) or as proportion. Due to the data distribution, non-parametric tests were used for group comparison. The Kruskal–Wallis test was used for continuous variables and the χ^2^-test for binary variables.

To describe demographic and diabetes-related outcomes between the T1D + PTSD group and the T1D-no-PTSD group as well as to the T1D-only group the variables gender, migration background, age in years, height in cm, BMI, age at diabetes onset, diabetes duration, type of insulin treatment (injection vs. pump therapy), continuous glucose measurement (CGM; ≥ 1 day), HbA1c (in % and mmol/mol), daily insulin dose (U/kg body weight) were analyzed. The following additional mental comorbidities were compared between the T1D + PTSD group and the T1D-no-PTSD group: depression, anxiety/obsessive–compulsive disorder, attention and activity disorder, eating disorder, borderline personality disorder, and suicidality. Anorexia nervosa, bulimia nervosa, binge eating and eating disorder not otherwise specified were subsumed under the term ´eating disorders`. All mental disorders were defined as documented diagnosis in DPV according to DSM-5 and ICD-10 codes.

Patients with T1D and comorbid PTSD were matched 1:5 {variables: age (continuously and in groups: ≤ 25; > 25 years), gender, diabetes duration (continuously and in groups: ≤ 2; 2–5; > 5–10; > 10–20; > 20 years), type of insulin treatment (injection vs. pump therapy), migration background} with patients of the T1D-no-PTSD group. Equally, patients with T1D and comorbid PTSD were matched in the same ratio with the same variables with patients in the T1D-only group. Matching was conducted (greedy-matching algorithm) with a caliper width of 0.2. Standardized differences were assessed before and after matching to evaluate balancing of covariates between the matched cohorts. For age groups ≤ 25 and > 25 and the variable gender, we used exact matching to ensure that all patients belonged to the respective group.

We compared the T1D + PTSD and the matched T1D-no-PTSD group, but also the T1D + PTSD with the matched T1D-only group regarding the variables BMI-SDS (AGA), HbA1c and daily insulin dose. For the number of hospital days (per patient-year), the hospital admissions (per patient-year), the rate of DKA (per patient-year) and the rate of severe hypoglycemia (per person-year) with or without coma we assumed a negative binomial distribution. All analyses were performed separately for each of the age groups ≤ 25 and > 25 years, since the definition of adolescence up to the age of 24 years corresponds to the current growth patterns of adolescents and the general understanding of this phase of life^32^. Statistical significance was assumed for p value < 0.05 (two-tailed) (Supplementary Information).

## Results

**Figure 1:**
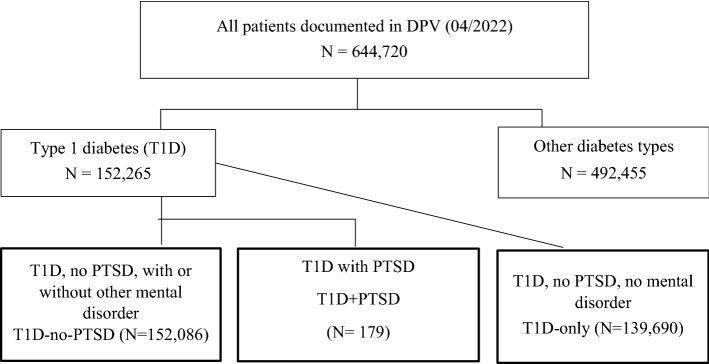
**Selection of study population and group information**. Flow chart. *T1D* type 1 diabetes, *DPV* diabetes prospective follow-up, *PTSD* posttraumatic stress disorder, *T1D-no-PTSD* patients with T1D, with or without other mental comorbidities, not including PTSD, *T1D + PTSD* T1D patients with PTSD, with or without other mental comorbidities, *T1D-only* T1D patients without mental comorbidities, no PTSD.

A total of 644,720 patients were documented in the DPV registry, of whom 152,265 were classified as T1D. 179 patients with documented comorbid PTSD (with or without other mental health comorbidities) in addition to T1D formed the T1D + PTSD group. 152,086 patients, without documented PTSD (with or without other mental health comorbidities) formed the T1D-no-PTSD group. This T1D-no-PTSD group consists of all remaining T1D patients who have not documented a PTSD diagnosis. 139,690 patients had no documentation of any comorbid mental disease and therefore also no PTSD, these patients represent the T1D-only group (Fig. 1).

**Figure 2:**
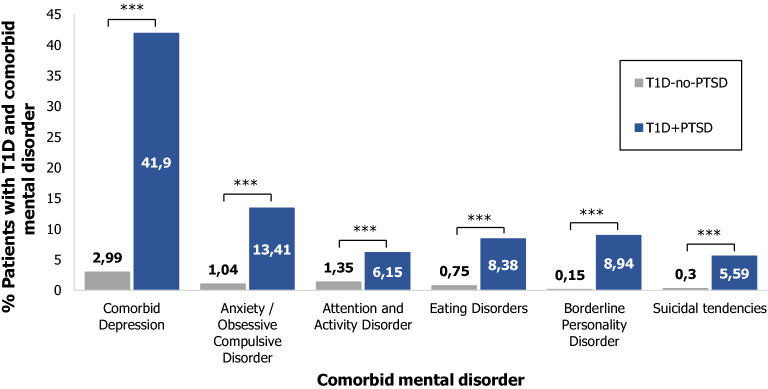
**Mental comorbidities of patients with T1D and comorbid PTSD, compared to patients with T1D and no comorbid PTSD**. All data are presented in percentage [%]. *T1D* type 1 diabetes, *PTSD* posttraumatic stress disorder, *T1D + PTSD* patients with T1D and posttraumatic stress disorder, *T1D-no-PTSD* patients with or without other mental comorbidities, not including PTSD; **significant (p < 0.01); ***significant (p < 0.001).

Compared to the T1D-no-PTSD group, patients with T1D and comorbid PTSD were significantly more often diagnosed with other concomitant mental disorders (depression, anxiety/obsessive–compulsive disorder, attention and activity disorder, eating disorder, borderline personality disorder, and suicidality) (Table 1 and illustrated in Fig. 2).

**Table 1:** Baseline characteristics of patients with T1D in all groups.

Demographics, diabetes-related outcomes	T1D-only N = 139,690	T1D + PTSD N = 179	T1D-no-PTSD N = 152,086	*p *value (T1D-only vs. T1D + PTSD)	*p *value (T1D-no-PTSD vs. T1D + PTSD)
Gender [% male]	53.30	29.61	53.05	< 0.001*	< 0.001*
Migration background [%]	12.40	11.73	12.29	1.000	1.000
Age, in years	18.03 [14.99; 40.05]	25.85 [17.42; 46.15]	18.08 [15.16; 40.80]	< 0.001*	< 0.001*
Age at diabetes onset	11.64 [6.87; 20.64]	13.46 [7.23; 26.08]	11.65 [6.91; 20.82]	0.072	0.112
Diabetes duration, in years	7.53 [3.20; 14.34]	11.66 [6.48; 21.29]	7.70 [3.33; 14.54]	< 0.001*	< 0.001*
Height-SDS, AGA	− 0.18 [− 0.90; 0.52]	− 0.49 [− 1.30; 0.21]	− 0.18 [− 0.92; 0.51]	0.001*	< 0.01*
BMI-SDS, AGA	0.31 [− 0.47; 1.05]	0.58 [− 0.60; 1.30]	0.31 [− 0.48; 1.07]	0.602	0.655
Daily Insulin dose/kg	0.77 [0.57; 0.99]	0.70 [0.48; 0.93]	0.77 [0.57; 0.99]	0.101	0.119
Insulin pump [%]	35.81	36.87	36.00	1.000	1.000
CGM > 1/day [%]	28.98	36.87	29.25	0.101	0.119
HbA1c [%]	7.67 [6.86; 8.74]	8.02 [7.15; 9.02]	7.69 [6.88; 8.79]	0.060	0.119
HbA1c: [mmol/mol]	60.35 [51.52; 71.98]	64.19 [54.60; 75.06]	60.58 [51.66; 72.54]	0.060	0.119

Mental comorbidities	T1D-only	T1D + PTSD N = 179	T1D-no-PTSD N = 156,086		*p *value (T1D-no-PTSD vs. T1D + PTSD)
Comorbid depression [%]		41.90	2.99		< 0.001*
Anxiety/obsessive compulsive disorder [%]		13.41	1.04		< 0.001*
Attention and activity disorder [%]		6.15	1.35		< 0.001*
Eating disorders [%]		8.38	0.75		< 0.001*
Borderline personality disorder [%]		8.94	0.15		< 0.001*
Suicidal tendencies [%]		5.59	0.30		< 0.001*

All data are presented in median [25% quantile; 75% quantile] or percentage [%]. T1D = type 1 diabetes; PTSD = posttraumatic stress disorder ; T1D-only = patients with T1D without comorbid mental disorder; T1D + PTSD = patients with T1D and PTSD, with or without other mental comorbidities; T1D-no-PTSD = patients with or without other mental comorbidities, no PTSD; BMI = body-mass index; SDS = standard deviation score; AGA = Arbeitsgemeinschaft Adipositas im Kindes- und Jugendalter; CGM = continuous glucose monitoring; HbA1c = Hemoglobin A1c. * significant (p < 0.05).

In the T1D-no-PTSD group of patients ≤ 25 years before matching (n = 97,232), the distribution between men (53%) and women (47%) was balanced, while in the T1D + PTSD group of the same age group (n = 88), there were significantly more women (69%). Patients in the T1D + PTSD group were significantly older, had a higher BMI-SDS, and higher HbA1c (Table 2). Among patients > 25 years of age, 53% of the T1D-no-PTSD group (n = 54,854) were male. In the T1D + PTSD (n = 91) group were significantly more women (72%) then men. Patients in the T1D + PTSD group had a higher percentage of CGM (Table 2). In the T1D-only group of patients ≤ 25 years before matching (n = 89,819), the distribution between men (53%) and women (47%) was balanced. Patients in the T1D + PTSD group were significantly more women, older, had a longer duration of diabetes, a higher BMI-SDS, and higher HbA1c (Table 3). Among patients > 25 years of age, 54% of the T1D-only group (n = 49,871) were male. In the T1D + PTSD (n = 91) group were significantly more women (71%) then men (Table 3).

**Table 2:** Baseline characteristics of patients with T1D (for age ≤ 25, > 25 years) before and after matching (variables: age, gender, diabetes duration, type of insulin treatment and migration background), comparing patients with T1D and comorbid PTSD to patients with T1D without PTSD.

Demographics, diabetes-related outcomes	Entire age range	≤ 25 years			> 25 years		
	SMD	T1D-no-PTSD N = 97,232	T1D-PTSD N = 88	*p *value	T1D-no-PTSD N = 54,854	T1D-PTSD N = 91	*p *value
Characteristics before matching							
Overall Propensity Score	0.78						
Gender [% male]	− 0.50	53.16	30.68	< 0.001*	52.85	28.57	< 0.001*
Migration background [%]	0.10	18.42	22.73	1.000	1.44	1.10	1.000
Age, in years	0.47	16.47 [12.84; 17.83]	17.41 [15.37; 18.66]	< 0.01*	50.62 [37.70; 63.11]	45.78 [37.29; 61.26]	1.000
Age at diabetes onset		8.97 [5.26; 12.30]	9.70 [5.55; 12.80]	1.000	29.45 [17.15; 43.28]	26.07 [14.55; 38.94]	0.836
Diabetes duration, in years	0.42	5.63 [2.51; 9.46]	7.56 [4.15; 11.03]	0.015	17.93 [7.98; 29.61]	21.03 [12.69; 32.31]	0.722
Height-SDS, AGA		− 0.07 [− 0.77; 0.62]	− 0.55 [− 1.49; 0.35]		− 0.40 [− 1.27; 0.29]	− 0.49 [− 1.20; 0.13]	
BMI-SDS, AGA		0.52 [− 0.15; 1.20]	1.01 [0.11; 1.62]	0.017*	− 0.22 [− 1.06; 0.68]	− 0.23 [− 1.27; 0.95]	1.000
Daily Insulin dose/kg		0.84 [0.66; 1.04]	0.82 [0.68; 1.05]	1.000	0.57 [0.42; 0.76]	0.52 [0.40; 0.70]	1.000
Insulin pump [%]	− 0.01	41.14	39.77	1.000	23.75	34.07	0.231
CGM > 1/day [%]		38.39	43.18	1.000	13.05	30.77	< 0.001*
HbA1c [%]		7.78 [6.97; 8.89]	8.32 [7.37; 9.45]	0.011*	7.53 [6.72; 8.59]	7.73 [6.71; 8.87]	1.000
HbA1c: [mmol/mol]		61.50 [52.67; 73.64]	67.41 [57.08; 79.78]	0.011*	58.77 [49.92; 70.33]	61.00 [49.83; 73.43]	1.000

Demographics, diabetes-related outcomes	Entire age range	≤ 25 years			> 25 years		
	SMD	T1D-no-PTSD N = 440	T1D-PTSD N = 88	*p *value	T1D-no-PTSD N = 455	T1D-PTSD N = 91	*p *value
Characteristics after matching							
Overall Propensity Score	0.00						
Gender [% male]	0.00	30.68	30.68	1.000	28.57	28.57	1.000
Migration background [%]	0.00	22.73	22.73	1.000	1.10	1.10	1.000
Age, in years	0.02	17.32 [15.10; 18.28]	17.41 [15.37; 18.66]	1.000	48.25 [37.17; 61.67]	45.78 [37.29; 61.26]	1.000
Age at diabetes onset		8.62 [5.35; 12.51]	9.70 [5.55; 12.80]	1.000	25.36 [14.63; 38.74]	26.07 [14.55; 38.94]	1.000
Diabetes duration, in years	− 0.02	7.59 [4.41; 11.05]	7.56 [4.15; 11.03]	1.000	20.06 [12.31; 30.79]	21.03 [12.69; 32.31]	1.000
Height-SDS, AGA		− 0.13 [− 0.80; 0.57]	− 0.55 [− 1.49; 0.35]		− 0.49 [− 1.27; 0.29]	− 0.49 [− 1.20; 0.13]	
BMI-SDS, AGA		0.63 [− 0.05; 1.31]	1.01 [0.11; 1.62]	0.616	− 0.13 [− 0.96; 0.69]	− 0.23 [− 1.27; 0.95]	1.000
Daily Insulin dose/kg		0.87 [0.68; 1.09]	0.82 [0.68; 1.05]	1.000	0.59 [0.43; 0.77]	0.52 [0.40; 0.70]	1.000
Insulin pump [%]	0.00	39.77	39.77	1.000	34.07	34.07	1.000
CGM > 1/day [%]		40.68	43.18	1.000	19.12	30.77	0.167
HbA1c [%]		7.98 [7.14; 9.17]	8.32 [7.37; 9.45]	0.759	7.47 [6.70; 8.56]	7.73 [6.71; 8.87]	1.000
HbA1c: [mmol/mol]		63.71 [54.48; 76.71]	67.41 [57.08; 79.78]	0.759	58.19 [49.74; 70.07]	61.00 [49.83; 73.43]	1.000

All data are presented in median [Q1; Q3] or percentage [%]. Entire age range = Total age range of patients in the T1D + PTSD group and T1D-only group. *SMD* standardized mean difference between T1D-no-PTSD and T1D + PTSD group, *T1D* type 1 diabetes, *PTSD* posttraumatic stress disorder, *T1D-no-PTSD* patients with T1D without PTSD, with or without other comorbid mental disorder, *T1D + PTSD* patients with T1D and PTSD, with or without other mental comorbidity, *BMI* body-mass index, *SDS* standard deviation score, *AGA* Arbeitsgemeinschaft Adipositas im Kindes- und Jugendalter, *CGM* continuous glucose monitoring, *HbA1c* Hemoglobin A1c.

*Significant (p < 0.05).

**Table 3:** Baseline characteristics of patients with T1D (for age ≤ 25, > 25 years) before and after matching (variables: age, gender, diabetes duration, type of insulin treatment and migration background), comparing patients with T1D and comorbid PTSD to patients with T1D and no comorbid mental disorder.

Demographics, diabetes-related outcomes	Entire age range	≤ 25 years			> 25 years		
	SMD	T1D-only N = 89,819	T1D + PTSD N = 88	*p *value	T1D-only N = 49,871	T1D + PTSD N = 91	*p value*
Characteristics before matching							
Overall propensity score	0.79						
Gender [% male]	− 0.5	52.93	30.68	< 0.001*	53.95	28.57	< 0.001*
Migration background [%]	0.10	18.50	22.73	1.000	1.41	1.10	1.000
Age, in years	0.48	16.39 [12.62; 17.82]	17.41 [15.37; 18.66]	< 0.001*	50.38 [37.42; 63.04]	45.78 [37.29; 61.26]	1.000
Age at diabetes onset		8.95 [5.23; 12.32]	9.70 [5.55; 12.80]	0.920	29.50 [17.27; 43.32]	26.07 [14.55; 38.94]	0.770
Diabetes duration, in years	0.44	5.51 [2.40; 9.34]	7.56 [4.15; 11.03]	< 0.01*	17.70 [7.71; 29.33]	21.03 [12.69; 32.31]	0.501
Height-SDS, AGA		− 0.06 [− 0.76; 0.62]	− 0.55 [− 1.49; 0.35]		− 0.40 [− 1.27; 0.29]	− 0.49 [− 1.20; 0.13]	
BMI-SDS, AGA		0.51 [− 0.14; 1.18]	1.01 [0.11; 1.62]	0.014*	− 0.23 [− 1.07; 0.66]	− 0.23 [− 1.27; 0.95]	1.000
Daily insulin dose/kg		0.84 [0.66; 1.04]	0.82 [0.68; 1.05]	1.000	0.57 [0.42; 0.76]	0.52 [0.40; 0.70]	1.000
Insulin pump [%]	− 0.01	40.89	39.77	1.000	23.51	34.07	0.195
CGM > 1/day [%]		38.01	43.18	1.000	12.72	30.77	< 0.001*
HbA1c [%]		7.75 [6.96; 8.81]	8.32 [7.37; 9.45]	< 0.01*	7.50 [6.70; 8.58]	7.73 [6.71; 8.87]	1.000
HbA1c: [mmol/mol]		64.23 [52.52; 72.82]	67.41 [57.08; 79.78]	< 0.01*	58.49 [49.73; 70.22]	61.00 [49.83; 73.43]	1.000

Demographics, diabetes-related outcomes	Entire age range	≤ 25 years			> 25 years		
	SMD	T1D-only N = 440	T1D + PTSD N = 88	*p value*	T1D-only N = 455	T1D + PTSD N = 91	*p value*
Characteristics after matching							
Overall propensity score	0.00						
Gender [% male]	0.00	30.68	30.68	1.000	28.57	28.57	1.000
Migration background [%]	0.00	22.73	22.73	1.000	1.10	1.10	1.000
Age, in years	0.02	17.22 [14.43; 18.21]	17.41 [15.37; 18.66]	0.939	49.43 [35.60; 61.59]	45.78 [37.29; 61.26]	1.000
Age at diabetes onset		8.67 [4.96; 11.96]	9.70 [5.55; 11.80]	1.000	25.29 [13.18; 38.48]	26.07 [14.55; 38.94]	1.000
Diabetes duration, in years	− 0.01	7.63 [4.42; 11.04]	7.56 [4.15; 11.03]	1.000	21.04 [12.31; 31.44]	21.03 [12.69; 32.31]	1.000
Height-SDS, AGA		− 0.10 [− 0.88; 0.54]	− 0.55 [− 1.49; 0.35]	0.054	− 0.49 [− 1.27; 0.29]	− 0.49 [− 1.20; 0.13]	1.000
BMI-SDS, AGA		0.61 [− 0.02; 1.31]	1.01 [0.11; 1.62]	1.000	− 0.25 [− 0.99; 0.62]	− 0.23 [− 1.27; 0.95]	1.000
Daily insulin dose/kg		0.85 [0.68; 1.05]	0.82 [0.68; 1.05]	1.000	0.57 [0.41; 0.77]	0.52 [0.40; 0.70]	1.000
Insulin pump [%]	0.00	39.77	39.77	1.000	34.07	34.07	1.000
CGM > 1/day [%]		40.68	43.18	1.000	12.09	30.77	< 0.001*
HbA1c [%]		8.08 [7.20; 9.05]	8.32 [7.37; 9.45]	0.080	7.59 [6.77; 8.67]	7.73 [6.71; 8.87]	1.000
HbA1c: [mmol/mol]		64.81 [55.20; 75.45]	67.41 [57.08; 79.78]	0.080	59.50 [50.44; 71.27]	61.00 [49.83; 73.43]	1.000

All data are presented in median [Q1; Q3] or percentage [%]. Entire age range = Total age range of patients in the T1D + PTSD group and T1D-only group. *SMD* standardized mean difference between T1D-only group and T1D + PTSD group, *T1D* type 1 diabetes, *PTSD* posttraumatic stress disorder, *T1D-only* patients with T1D without comorbid mental disorder, *T1D + PTSD* patients with T1D and PTSD, with or without other mental disorder, *BMI* body-mass index, *SDS* standard deviation score, *AGA* Arbeitsgemeinschaft Adipositas im Kindes- und Jugendalter, *CGM* continuous glucose monitoring, *HbA1c* Hemoglobin A1c.

*Significant (p < .05).

The T1D control group and the T1D-no-PTSD group, stratified by age group, were matched 5:1 with the 179 patients who have comorbid PTSD documentation in addition to T1D. For each patient in the T1D + PTSD group, a matching partner from the T1D-no-PTSD and the T1D-only group was found and thus all patients in the T1D + PTSD group could be included in the further analysis even after matching. The standardized mean difference (SMD) of the general propensity score was SMD = 0.78 for T1D + PTSD/T1D-no-PTSD and SMD = 0.79 for T1D + PTSD/T1D-only before matching and SMD = 0.00 for both after matching. For detailed information on the data before and after matching, see Tables 2 and 3. The final sample is 1074 patients with whom the group comparisons were calculated.

The group comparison showed that patients in the T1D + PTSD group aged ≤ 25 years had significantly higher HbA1c {8.71 (T1D + PTSD) vs. 8.30 (T1D-no-PTSD); p ≤ 0.046; 8.71 (T1D + PTSD) vs. 8.24 (T1D-only); p ≤ 0.013}, more hospital admissions (0.93 vs. 0.44 per patient-year; p < 0.001; 0.93 vs. 0.32; p < 0.001) and higher rates of DKA (0.10 vs. 0.02 events/patient-year; p ≤ 0.001; 0.10 vs. 0.01 events/patient-year; p ≤ 0.001) than patients in the T1D-no-PTSD or T1D-only group. Patients in the T1D + PTSD group additionally had significantly higher numbers of hospital admissions due to DKA (0.36 vs. 0.08 per patient-year; p < 0.001; 0.36 vs. 0.05 per patient-year; p < 0.001), but also not due to DKA (0.61 vs. 0.37 per patient-year; p = 0.003; 0.61 vs. 0.28 per patient-year; p < 0.001), than patients in the T1D-no-PTSD or T1D-only group. The significantly higher rates of DKA in the T1D + PTSD group compared to the T1D-no-PTSD or T1D-only group remained after adjustment for overweight yes/no, defined as BMI ≥ 25 or BMI-SDS ≥ 1.28 (0.11 vs. 0.02 per year; p < 0.001; 0.11 vs. 0.01 per year; p < 0.001). Patients in T1D + PTSD group had significantly higher BMI (0.85 vs. 0.59; p = 0.046) and a longer hospital stay for inpatient admissions (15.89 vs. 11.58; p = 0.048), than patients in the T1D-no-PTSD group, but not in the T1D-only group (Table 4).

**Table 4:** Diabetes-related outcomes of patients with T1D (ages ≤ 25 and > 25 years) with PTSD compared to patients with T1D without PTSD (with or without other mental comorbidities) and compared to patients with T1D without a mental comorbidity, matched for demographics.

Diabetes-related outcomes	≤ 25 years					> 25 years				
	T1D + PTSD	T1D-only	*p value* (T1D + PTSD vs. T1D-only)	T1D-no-PTSD	*p value* (T1D + PTSD vs. T1D-no-PTSD)	T1D + PTSD	T1D-only	*p value* (T1D + PTSD vs. T1D-only)	T1D-no-PTSD	*p value* (T1D + PTSD vs. T1D-no-PTSD)
HbA1c, [%]	8.71 [8.37; 9.04]	8.24 [8.08; 8.39]	0.013*	8.30 [8.14; 8.47]	0.046*	7.92 [7.56; 8.29]	7.95 [7.78; 8.11]	0.915	7.88 [7.72; 8.05]	0.844
BMI-SDS, AGA	0.85 [0.62; 1.07]	0.63 [0.53; 0.73]	0.08	0.59 [0.49; 0.70]	0.046*	− 0.19 [− 0.46; 0.08]	− 0.15 [− 0.27; − 0.03]	0.773	− 0.13 [− 0.25; − 0.01]	0.693
Insulin dose/kg	0.91 [0.79; 1.03]	0.94 [0.89; 0.99]	0.665	0.91 [0.88; 0.95]	0.948	0.57 [0.49; 0.65]	0.62 [0.59; 0.66]	0.211	0.63 [0.59; 0.66]	0.114
Number of hospital days [n/py]^a^	15.89 [12.05; 20.98]	11.86 [9.94; 14.14]	0.08	11.58 [9.89; 13.57]	0.048*	20.35 [16.07; 25.78]	11.09 [10.15; 12.12]	< 0.001*	11.86 [10.79; 13.02]	< 0.001*
Number of hospital admissions [n/py]^a^	0.93 [0.72; 1.20]	0.32 [0.27; 0.38]	< 0.001*	0.44 [0.37; 0.51]	< 0.001*	0.47 [0.36; 0.63]	0.69 [0.62; 0.77]	0.015*	0.77 [0.70; 0.85]	0.003*
Number of hospital admissions due to Diabetic Ketoacidosis [n/py]^a^	0.36 [0.19; 0.71]	0.05 [0.03; 0.07]	< 0.001*	0.08 [0.05; 0.12]	< 0.001*	0.05 [0.02; 0.13]	0.10 [0.07; 0.15]	0.168	0.13 [0.09; 0.18]	0.071
Number of hospital admissions not due to Diabetic Ketoacidosis [n/py]^a^	0.61 [0.47; 0.81]	0.28 [0.23; 0.33]	< 0.001*	0.37 [0.31; 0.43]	0.003*	0.44 [0.33; 0.59]	0.64 [0.57; 0.71]	0.019*	0.71 [0.64; 0.79]	0.004*
Diabetic Ketoacidosis [n/py]^a^	0.10 [0.05; 0.21]	0.01 [0.01; 0.03]	< 0.001*	0.02 [0.01; 0.04]	< 0.001*	0.01 [0.00; 0.07]	0.00 [0.00; 0.01]	0.238	0.01 [0.01; 0.03]	0.978
Diabetic Ketoacidosis adjusted for BMI [n/py]^a^	0.11 [0.05; 0.23]	0.01 [0.01; 0.03]	< 0.001*	0.02 [0.01; 0.04]	< 0.001*	0.01 [0.00; 0.08]	0.00 [0.00; 0.02]	0.245	0.01 [0.01; 0.03]	0.980
Severe Hypoglycemia [n/py]^a^	0.13 [0.04; 0.38]	0.20 [0.13; 0.31]	0.444	0.15 [0.09; 0.24]	0.792	0.13 [0.04; 0.39]	0.19 [0.11; 0.30]	0.551	0.19 [0.13; 0.28]	0.484
Hypoglycemic coma [n/py]^a^	0.05 [0.01; 0.16]	0.02 [0.01; 0.04]	0.264	0.03 [0.02; 0.06]	0.545	0.02 [0.01; 0.11]	0.06 [0.04; 0.10]	0.233	0.06 [0.04; 0.10]	0.246

All data are presented in: estimated mean [lower confidence interval; upper confidence interval] of the matched cohort (matching variables: age, gender, diabetes duration, therapy and migration background). *T1D* type 1 diabetes, *PTSD* posttraumatic stress disorder, *T1D-only* patients with T1D without comorbid mental disorder, *T1D + PTSD* patients with T1D and PTSD, with or without other mental disorder, *T1D-no-PTSD* patients with T1D without PTSD, with or without other comorbid mental disorder, *HbA1c* Hemoglobin A1c, *BMI* body-mass index, *SDS* standard deviation score, *AGA* Arbeitsgemeinschaft Adipositas im Kindes- und Jugendalter; ^a^Number of events per patient years.

*Significant (p < 0.05).

Patients in the T1D + PTSD group over 25 years had significantly fewer hospital admissions {0.47 (T1D + PTSD) vs. 0.77 (T1D-no-PTSD) per patient-year; p = 0.003; 0.47 (T1D + PTSD) vs. 0.69 (T1D-only} per patient-year; p < 0.015), but longer hospital stay for inpatient admissions (20.35 vs. 11.86 days; p < 0.001; 20.35 vs. 11.09 days; p < 0.001) than patients in the T1D-no-PTSD or T1D-only group. This was only true for hospital admissions not due to DKA (0.44 vs. 0.71 per person-year; p = 0.004; 0.44 vs. 0.64 per person-year; p = 0.019) in the T1D + PTSD group compared with patients in the T1D-no-PTSD or T1D-only group. The remaining diabetes-related outcomes showed no significant differences between T1D + PTSD and T1D-no-PTSD or T1D-only group in both age groups (Table 4).

## Discussion

To our knowledge, there has been no study to date that has examined multiple diabetes-related outcomes in patients with T1D and comorbid PTSD compared to patients with T1D without PTSD and to patients with T1D without other comorbid mental disorder across the lifespan (but focusing on different age groups). The study results showed that comorbid PTSD was associated with worse diabetes-related outcomes such as DKA, HbA1c and with higher number of hospital admissions in patients with T1D aged 25 years or younger compared to patients with T1D without PTSD or without a comorbid mental disorder. In addition, CAYA with T1D and comorbid PTSD were found to have higher BMI and longer hospital stays compared to CAYA with T1D without PTSD. These results, which highlight the negative association between PTSD diagnosis and diabetes-related outcomes, clearly show how relevant PTSD is in clinical practice for the disease progression of young patients with T1D and that awareness should be raised.

The demographic findings that more women with T1D were in the sample with comorbid PTSD confirms epidemiologic evidence that women are significantly more likely to be affected by PTSD than men (2–3:1)^33^. Reasons for this, besides personal, social and cultural factors, include the fact that women experience more severe trauma (e.g. sexual trauma) than men, and at a younger age. Trauma at a young age has a higher impact, especially if it is a type II trauma (complex trauma such as abuse or torture)^33,34^. The gender differences in the development of PTSD are due to the stronger perception of threat and loss of control in women during trauma, a higher level of peri-traumatic dissociation, the sex-specific acute psychobiological reactions to trauma and the more emotion-oriented, defensive and palliative coping style to deal with trauma-related symptoms compared to men^33,34^. In summary, female patients with T1D are a particularly vulnerable group for comorbid PTSD. Since studies have shown that women benefit more from psychotherapy than men in reducing PTSD^33^, increased attention to this at-risk group, psychosocial diagnostics and referral to psychotherapeutic treatment when necessary may be promising in reducing this comorbidity. In the DPV documentation, patients with T1D and comorbid PTSD had significantly more mental comorbidities than all other patients with T1D. This result is also reflected in the frequency distribution of the general population. Although mental disorders are common, severe cases are often concentrated in a small proportion with multiple mental comorbidities^35^.

Patients with T1D older than 25 years with comorbid PTSD showed less hospital admissions, but a longer inpatient hospital stay than patients with T1D without PTSD or without a comorbid mental disorder. The type of institution might be a reason for the longer inpatient hospital stay not due to DKA for patients with T1D and comorbid PTSD compared to patients with T1D without PTSD or without comorbid mental disorder. The average inpatient psychiatric or psychotherapeutic length of stay for the treatment of mental disorders is substantially longer than the length of stay for the treatment of a patient with T1D (25 vs. 7 days)^36,37^. As psychotherapeutic interventions to improve treatment adherence or reduce symptoms of comorbid mental disorders (such as depression) may have a positive effect on HbA1c levels in addition to the primary treatment goal^38,39^, it is possible that patients with T1D and PTSD may even have a positive impact on T1D disease progression, such as the number of hospital admissions, as a result of treatment for the comorbid mental disorder compared to patients without psychological support. Furthermore, patients with T1D and PTSD may have learned to better adjust their CD due to a longer hospital stay and consistent good medical care during hospitalization. An improvement in T1D self-management could also be a reason for a lower number of hospital admissions. Especially since initial results indicate that short-term hospitalization may not be the right occasion to intervene in the long-term diabetes management and achieve improvements^40^.

Adequate treatment and good glycemic control, reflected in low HbA1c levels, significantly reduce the risk of long-term vascular and neurological complications^41–44^. Conversely, this means that the significantly higher HbA1c level of CAYA with T1D and comorbid PTSD compared to CAYA with T1D without PTSD or without other mental comorbidities carries a high long-term health risk and can have a lasting impact on the further life of the still young patients with T1D. Severe, acute hyperglycemia may lead to DKA. DKA are potentially life-threatening complications that require intensive medical treatment and can have a significant impact on the patient and his or her social environment. DKA requires medical treatment, which partly explains the increased hospital admissions in addition to the increased DKA episodes in the CAYA with comorbid PTSD. DKA episodes and hospital admissions for T1D with early onset are among the categories that generate the highest healthcare costs. Female gender, pubertal age and poor glycemic control were associated with higher total costs^45^.

The findings that CAYA with T1D and comorbid PTSD had higher BMI and longer hospital stays compared to CAYA with T1D without PTSD, but not compared to CAYA without other comorbid mental disorder, may be indicative of the PTSD specific impact on diabetes-related outcomes. It is well-established that PTSD is associated with a reduced healthy diet and physical activity, as well as increased obesity^16^. The co-existence of obesity at a young age can have a negative clinical impact on all stages of diabetes course, which in turn could induce prolonged hospital stay^46^.

Adults had a substantially longer duration of T1D (8 vs. 20 years) and thus a higher T1D experience than CAYA. Adult patients with T1D older than 30 years had lower glucose variability/higher glycemic stability compared to adults younger than 30 years^47^. Stability in midlife and old age, as well as therapy that has become a constant habit, could reduce diabetes-specific emotional distress^22^. Whereas this diabetes-specific distress in turn moderates the occurrence of comorbid mental disorders, metabolic outcomes and well-being^48^. In addition, lack of family support and parental involvement in T1D treatment may lead to deteriorating glycemic control (i.e. higher HbA1c levels) in CAYA^49,50^. Due to developmental milestones achieved, adult patients usually do not need this type of support^22,51^. These aspects may explain why CAYA with T1D and comorbid PTSD have increased HbA1c and DKA compared with CAYA with T1D without PTSD or without other mental disorder, but adults do not have the worsening in metabolic outcomes and a better glycemic control.

CAYA with T1D and comorbid anxiety disorder also had increased HbA1c, DKA and hospital admissions compared with controls^18^. This association could be due to symptom overlap such as anxiety, psychological and physiological/autonomic hyperarousal (for example, exaggerated startle response, sleep disturbances, irritability, difficulty concentrating and excessive alertness) and avoidance behavior of anxiety disorders and PTSD ^3^. The autonomic hyperarousal in anxiety and PTSD is similarly manifest in acute or prolonged distress and can lead to insulin resistance and hyperglycemia^22^. These diabetes-related consequences of autonomic hyperarousal could be prevented by anticipatory diabetes self-management. As physiological responses to distress are rarely predictable, there are no specific response patterns that a patient with T1D could rely on^22^. In addition, the accuracy of perception of internal body processes (interoception) is important, especially hyperarousal. However, there is evidence from the literature that stress-related interoception is impaired in individuals with mental disorders^52^ or individuals with childhood trauma^53^. As well, avoidance behavior is a symptom in anxiety disorders, but also in PTSD^3^. If insulin treatment leads to confrontation with anxiety (in anxiety disorder) or circumstances similar to or related to the trauma (in PTSD triggered by medical trauma), the insulin treatment could be more likely to be avoided in these patients than in CAYA without mental disorders^25–27^. Avoidance of insulin treatment can lead to irregular and inadequate insulin control and treatment, which in turn can result in increased DKA, HbA1c and hospital admissions.

 Only 28.8% of adolescents with mental disorder in Germany are in treatment^54^ and this despite the fact that the effectiveness (e.g. reduction of mental disorder symptoms, increase of self-efficacy, increased glycemic control and self-management) of behavioral interventions in CAYA with T1D is well proven^55^. Lack of (additional) psychological care may maintain the poorer diabetes-related outcomes of CAYA with T1D and comorbid PTSD compared to CAYA with T1D without PTSD or without other mental comorbidities.

### Strengths and limitations

A limitation is that due to the cross-sectional design of this study, it was not possible to make causal statements about PTSD and diabetes-related outcomes. Another limitation is that the DPV documentation does not have information on the timing or type of trauma, the timing of diagnosis or duration and severity of PTSD or information on the treatment of comorbid PTSD. Treatment centers differ in their focus on mental comorbidity, resulting in different rates of documented comorbid mental disorders. As the general detection rate for mental disorders is also only 50%^56^, mental comorbidities in patients with T1D may have been underestimated in this study. A strength of the study is that the data of patients with T1D and comorbid PTSD were analyzed comparatively with two control groups and thus an interpretation of the results is possible for the diagnosis of PTSD, but also for other mental disorders. Another strength is that the search strings for the documentation in the DPV were conducted according to the documented diagnosis of PTSD to avoid bias in the results. However, this does not enable conclusions to be made about patients with T1D and comorbid PTSS in relation to diabetes-related outcomes. Since even elevated PTSS can have a significant negative impact on chronic disease severity, treatment adherence, health problems and functional impairment^57–59^, this would be an interesting future research question.

## Conclusions

CAYA with T1D have the strongest negative impact due to comorbid PTSD on diabetes-related outcomes HbA1c, DKA, BMI, hospital admission and length of hospital stay. These consequences, in the context of the increased prevalence of PTSD in T1D^5,6^ compared to adolescents without T1D^4^, point to the relevance and importance of this comorbidity in clinical practice. It should therefore be considered to screen this at-risk group preventively for PTSD or psychological distress in routine healthcare. Early detection and psychological counseling- ideally through the early involvement of a multidisciplinary diabetes specialist team—are necessary^60,61^.

## Supplementary Information

Supplementary Information.

## Data Availability

In order to ensure patient protection and to comply with the available consent forms, patient level data cannot be transferred outside of the Institute for Epidemiology and Medical Biometry, University of Ulm. However, joint research projects or remote data processing are possible. Further information about the data and conditions for access are available at https://www.d-p-v.eu/datenschutz.
